# Factors associated with inability to access addiction treatment among people who inject drugs in Vancouver, Canada

**DOI:** 10.1186/s13011-016-0053-6

**Published:** 2016-02-25

**Authors:** Amy Prangnell, Ben Daly-Grafstein, Huiru Dong, Seonaid Nolan, M-J Milloy, Evan Wood, Thomas Kerr, Kanna Hayashi

**Affiliations:** British Columbia Centre for Excellence in HIV/AIDS, St. Paul’s Hospital, 608-1081 Burrard Street, Vancouver, BC V6Z 1Y6 Canada; Human Biology Program, University of Toronto, Wetmore Hall, Room 105, 300 Huron Street, Toronto, ON M5S 3J6 Canada; Department of Medicine, University of British Columbia, St. Paul’s Hospital, 608-1081 Burrard Street, Vancouver, BC V6Z 1Y6 Canada

**Keywords:** Injection drug use, Addiction treatment, Homelessness, Drug or alcohol treatment, Binge drug use, Violence

## Abstract

**Background:**

Addiction treatment is an effective strategy used to reduce drug-related harm. In the wake of recent developments in novel addiction treatment modalities, we conducted a longitudinal data analysis to examine factors associated with inability to access addiction treatment among a prospective cohort of persons who inject drugs (PWID).

**Methods:**

Data were derived from two prospective cohorts of PWID in Vancouver, Canada, between December 2005 and November 2013. Using multivariate generalized estimating equations, we examined factors associated with reporting an inability to access addiction treatment.

**Results:**

In total, 1142 PWID who had not accessed any addiction treatment during the six months prior to interview were eligible for this study, including 364 women (31.9 %). Overall, 188 (16.5 %) reported having sought but were ultimately unsuccessful in accessing addiction treatment at least once during the study period. In multivariate analysis, factors independently and positively associated with reporting inability to access addiction treatment included: binge drug use (Adjusted Odds Ratio [AOR] = 1.65), being a victim of violence (AOR = 1.77), homelessness (AOR = 1.99), and having ever accessed addiction treatment (AOR = 2.33); while length of time injecting was negatively and independently associated (AOR = 0.98) (all *p* < 0.05).

**Conclusions:**

These findings suggest that sub-populations of PWID were more likely to report experiencing difficulty accessing addiction treatment, including those who may be entrenched in severe drug addiction and vulnerable to violence. It is imperative that additional resources go into ensuring treatment options are readily available when requested for these target populations.

## Background

Injecting illegal drugs remains a major public health concern with current estimates of 16 million users worldwide [1]. People who inject drugs (PWID) have a higher probability of contracting infectious diseases including HIV, bear an increased burden of morbidity and mortality, and may suffer from social isolation and stigma [1–3]. However, the risk of many of the negative consequences of injection drug use can be reduced by evidence-based addiction treatment [2], leading to a decrease in drug use, HIV risk and criminal behavior as well as increased likelihood of optimal HIV/AIDS treatment outcomes [4–6].

Now more than ever, a range of novel evidence-based addiction treatment modalities have been developed, including the expanded availability of methadone and use of both buprenorphine and buprenorphine/naloxone for opiate addiction as well as residential treatment and outpatient treatment [7, 8]. In Vancouver, Canada, which is home to Canada’s largest street-based drug scene [9], publically funded addiction treatment programs include opiate agonist therapy, residential and outpatient treatment and inpatient and outpatient withdrawal management, and have recently been increasingly made available [10, 11].

In many settings, despite various addiction prevention and care initiatives in place, certain populations of people who use drugs have previously been shown to experience increased barriers to access addiction treatment, including Indigenous peoples and persons with disabilities [12, 13]. Other barriers to treatment experienced by potential patients range from responsibility for child care, having negative attitudes towards drug treatment staff, and experiencing financial constraints, to the fear and potential stigmatization of being labelled a drug user [14–16]. Of concern, those who were unable to access addiction treatment have been shown to have higher rates of HIV risk behaviors and subsequent seroconversion to HIV [17, 18]. For this reason, those PWID who are unable to access addiction treatment may be among the most vulnerable populations.

Given the well documented benefits of addiction treatment and the serious consequences arising when barriers to treatment exist, identifying drug-using populations that experience an inability to access treatment is important. Doing so will identify patients who may benefit from targeted interventions to increase access to addiction treatment and thus improve their overall health. In the wake of recent developments in novel addiction treatment modalities, we sought to identify factors independently associated with an inability to access addiction treatment amongst PWID in a Canadian setting.

## Methods

### Study procedures

The Vancouver Injection Drug Users Study (VIDUS) and the AIDS Care Cohort to evaluate Exposure to Survival Services (ACCESS) are ongoing open prospective cohorts of adult drug users recruited through self-referral and street outreach in Vancouver, Canada. These studies have been described in detail previously [19]. Briefly, VIDUS enrolls HIV-negative persons who reported injecting an illicit drug at least once in the month preceding enrollment; ACCESS enrolls HIV-infected individuals who report using an illicit drug other than marijuana in the previous month. For both cohorts, other eligibility criteria included being aged 18 years or older, residing in the greater Vancouver region and providing written informed consent. The questionnaire provided is in English only, however migrants or foreigners are able to participate, provided they spoke English and reside in the Greater Vancouver region. Any VIDUS participants who seroconverted to HIV during follow-up were transferred to the ACCESS cohort so that VIDUS includes HIV-negative individuals only, and ACCESS includes HIV-positive individuals only. The study instruments and all other follow-up procedures for each study are essentially identical to allow for combined analyses. At baseline and semi-annually thereafter, participants complete the same interviewer-administered questionnaire eliciting sociodemographic data as well as information pertaining to drug use patterns, risk behaviors, and health care utilization. Nurses collect blood samples for HIV and Hepatitis C virus serology, provide basic medical care and arrange referrals to appropriate health care services if required. Participants receive a $30 (CDN) honorarium for each study visit. The University of British Columbia/Providence Healthcare Research Ethics Board provided ethical approval for both studies.

## Study sample and primary outcome measure

All participants who were enrolled in the cohorts between December 1, 2005 and November 30, 2013, and who reported injecting drugs in the six months preceding baseline were included in the present analysis. Additionally, at each follow up, the sample was restricted to individuals who did not report being enrolled in any addiction treatment in the previous six months. The primary outcome of interest was inability to access addiction treatment in the previous six months. This was defined as responding “yes” to the question: “In the past 6 months, have you tried to access any treatment program but were unable?” The same question has been used in a previous study, showing its criterion validity and reliability [17]. In the same questionnaire, participants were also asked about types of addiction treatment that they were unable to access, which included inpatient and outpatient detoxification services; residential treatment and recovery houses; outpatient treatment through community clinics offering opioid agonist treatment with methadone or buprenorophine/naloxone and addiction counseling; and twelve-step programmes (i.e., Narcotics/Cocaine/Alcoholics Anonymous). Participants were also asked to identify the reasons for why they were unable to access addiction treatment in an open-ended question.

### Study variables

Based on the literature, we selected explanatory variables that we hypothesized might be associated with having difficulty accessing addiction treatment [17, 20, 21]. These included sociodemographic data, including: age (per year older); gender (female vs. male); Caucasian (yes vs. no); homelessness in the previous six months (yes vs. no); employment in the previous six months (any employment vs. none); involvement in drug dealing in the previous six months (yes vs. no); involvement in sex work in the previous six months (yes vs. no); education attainment (high school completion or higher vs. less than high school). Drug-use variables referred to behaviours in the previous six months, and included: ≥ daily injection cocaine use (yes vs. no); ≥ daily injection heroin use (yes vs. no); ≥ daily injection crystal methamphetamine use (yes vs. no); ≥ daily injection prescription opioid use (yes vs. no); ≥ daily crack smoking (yes vs. no); ≥ daily alcohol use (yes vs. no); and binge drug use, defined as compulsive high-intensity injection drug use that exceeds normal patterns of consumption (yes vs. no) [22]. Other variables included: length of time since initiation of injection drug use (per year longer); having ever enrolled in drug or alcohol treatment (yes vs. no); experiencing an overdose in the previous six months (yes vs. no); being a victim of violence, defined as having been attacked, assaulted, or suffered violence in the previous six months (yes vs. no); being HCV antibody positive (yes vs. no); HIV status (being HIV infected and not receiving ART in the previous six months vs. being HIV infected and receiving ART in the previous six months vs. HIV negative); and incarceration in the previous six months (yes vs. no). Since the only difference in the eligibility criteria between the cohorts was the HIV serostatus, the HIV serostatus variable was included to adjust for the cohort designation.

### Statistical analysis

As a first step, we examined the baseline sample characteristics stratified by reports of inability to access addiction treatment, using the Pearson’s Chi-squared test (for binary variables) and Mann-Whitney test (for continuous variables). Fisher’s exact test was used when one or more of the cells contained expected values less than or equal to five.

Since analyses of factors potentially associated with inability to access addiction treatment included serial measures for each participant, we used generalized estimating equation (GEE) with logit link, which provided standard errors adjusted by multiple observations per person using an exchangeable correlation structure. Therefore, data from every participant follow-up visit were considered in this analysis. As a first step, we used bivariate GEE analyses to determine factors associated with inability to access addiction treatment. Next, because our study aimed to identify the set of variables that best explain a higher odds of inability to access addiction treatment, we used an *a priori*-defined backward model selection procedure based on examination of quasilikelihood under the independence model criterion statistic (QIC) to fit a multivariate model. In brief, we first included all explanatory variables that were associated with inability to access addiction treatment at the level of *p <* 0.10 in bivariate analyses in a full model. After examining the QIC of the model, we removed the variable with the largest *p*-value and built a reduced model. We continued this iterative process and selected the multivariate model with the lowest QIC value [23].

In a sub-analysis, we used descriptive statistics to examine specific addiction treatment modalities that participants commonly reported being unable to access, and reasons why they were unable to access the treatment. All *p*-values are two sided. All statistical analyses were performed using SAS software version 9.3 (SAS, Cary, NC).

## Results

In total, 1142 participants were eligible for the present study. Among this sample, 364 (31.9 %) were women, 644 (56.4 %) self-reported Caucasian ancestry and the median age at baseline was 41.9 years (interquartile range [IQR] = 34.9–48.0). Overall, the 1142 individuals contributed 5946 observations to the analysis and the median number of follow-up visits was 3 (IQR: 1–8). Of the 1142 individuals, 188 (16.5 %) reported a total of 250 reports of inability to access addiction treatment giving an incidence density of 5.1 reports (95 % confidence interval [CI]: 4.3–6.1) per 100 person-years. The baseline characteristics of all participants stratified by reported inability to access treatment are presented in Table 1.

**Table 1:** Baseline sample characteristics, stratified by reporting inability to access addiction treatment in the past six months among PWID in Vancouver, Canada (*n* = 1142)

Characteristic	Inability to access addiction treatment^a^		Odds ratio (95 % CI)	*p -* value
	Yes n (%) 68 (6.0)	No n (%) 1074 (94.0)		
Age (median, IQR)	39 (32–44)	42 (35–48)		0.008
Female gender	24 (35.3)	340 (31.7)	1.18 (0.70–1.97)	0.533
Caucasian	40 (58.8)	604 (56.2)	1.11 (0.68–1.83)	0.677
Homelessness^a^	45 (66.2)	392 (36.5)	3.73 (2.19–6.35)	<0.001
Daily injection cocaine use^a^	11 (16.2)	101 (9.4)	1.85 (0.94–3.64)	0.071
Daily injection heroin use^a^	32 (47.1)	361 (33.6)	1.75 (1.07–2.86)	0.025
Daily injection meth use^a^	1 (1.5)	56 (5.2)	0.27 (0.04–1.98)	**0.200**
Daily injection prescription opioid use^a^	27 (39.7)	310 (28.9)	1.62 (0.98–2.67)	0.059
Daily crack smoking^a^	29 (42.7)	422 (39.3)	1.15 (0.70–1.89)	0.583
Daily alcohol use^a^	**6 (8.8)**	**87 (8.1)**	**1.11 (0.47–2.65)**	**0.806**
Years since first injection drug use (median, IQR)	17 (11–22)	19 (11–28)		0.084
Drug or alcohol treatment Ever	59 (86.8)	804 (74.9)	2.20 (1.08–4.50)	0.027
Employment^a^	21 (30.9)	258 (24.0)	1.41 (0.83–2.41)	0.202
Drug dealing^a^	36 (52.9)	335 (31.2)	2.48 (1.51–4.06)	<0.001
Sex work^a^	7 (10.3)	150 (14.0)	0.72 (0.32–1.60)	0.413
High school degree	40 (58.8)	500 (46.6)	1.64 (0.99–2.72)	0.051
Overdose^a^	6 (8.8)	66 (6.2)	1.48 (0.62–3.54)	**0.372**
Binge drug use^a,b^	35 (51.5)	400 (37.2)	1.77 (1.08–2.90)	0.021
A victim of violence^a^	26 (38.2)	230 (21.4)	2.25 (1.35–3.74)	0.002
HCV positive	58 (85.3)	941 (87.6)	0.78 (0.39–1.57)	0.490
HIV+ and not on ART^a^	**10 (14.7)**	**164 (15.3)**	**0.88 (0.44–1.77)**	**0.720**
HIV+ and on ART^a^	**9 (13.2)**	**203 (18.9)**	**0.64 (0.31–1.32)**	**0.225**
Incarceration^a^	20 (29.4)	187 (17.4)	1.97 (1.14–3.39)	0.013

Note: The *p*-value for variable age was obtained from Mann-Whitney test; exact mid *p*-values for daily injection meth use and overdose were obtained from Fisher’s exact test; and for other binary variables p-values were obtained from Chi-square test with degree of freedom = 1

*PWID* people who inject drugs, *CI* confidence interval, *IQR* interquartile range

^a^Denotes activities in the previous six months

^b^Refers to any route of consumption (i.e., sniffing, snorting, smoking or injecting)

Also at baseline, compared to ACCESS, VIDUS participants were more likely to be young, be Caucasian, inject heroin or prescription opioids at least daily, have a history of drug or alcohol treatment, have employment, engage in drug dealing, experience violence, while they were less likely to engage in binge drug use and be HCV-positive (all *p* < 0.05). There were 1988 observations from ACCESS and 3958 observations from the VIDUS cohort. Among ACCESS observations, 72 (3.6 %) involved a report of inability to access addiction treatment, while there were 178 (4.5 %) reports in VIDUS. There was no significant difference in these reports between the two cohorts (Chi-square test *p*-value = 0.113).

The results of the bivariate and multivariate GEE analyses of factors associated with reporting being unable to access addiction treatment are presented in Table 2. As shown, in the final multivariate model, factors that remained independently associated with inability to access addiction treatment included: homelessness (adjusted odds ratio [AOR] = 1.99, 95 % CI: 1.47–2.69), time since initiating injecting drug use (AOR = 0.98, 95 % CI: 0.97–1.00), having ever accessed drug or alcohol treatment (AOR = 2.33, 95 % CI: 1.47–3.68), binge drug use (AOR = 1.65, 95 % CI: 1.26–2.16), and being a victim of violence (AOR = 1.77, 95 % CI: 1.29–2.42).

**Table 2:** Bivariate and multivariate GEE analyses of factors associated with inability to access addiction treatment among PWID in Vancouver, Canada (*n* = 1142)

Characteristic	Unadjusted		Adjusted	
	Odds ratio (95 % CI)	*p -* value	Odds ratio (95 % CI)	*p -* value
Age				
(per year older)	0.97 (0.96–0.98)	<0.001		
Gender				
(female vs. male)	1.42 (1.03–1.95)	0.033	1.37 (0.98–1.92)	0.062
Ethnicity				
(Caucasian vs. other)	0.82 (0.60–1.12)	0.205		
Homelessness^a^				
(yes vs. no)	2.53 (1.92–3.33)	<0.001	1.99 (1.47–2.69)	<0.001
Daily injection cocaine use^a^				
(yes vs. no)	1.58 (1.04–2.41)	0.032		
Daily injection heroin use^a^				
(yes vs. no)	1.69 (1.24–2.31)	<0.001		
Daily injection meth use^a^				
(yes vs. no)	1.15 (0.61–2.15)	0.669		
Daily injection prescription opioid use^a^				
(yes vs. no)	1.51 (1.11–2.07)	0.010		
Daily crack smoking^a^				
(yes vs. no)	1.30 (0.98–1.74)	0.073		
Daily alcohol use^a^				
(yes vs. no)	1.37 (0.90–2.11)	0.144		
Length of time injecting drugs				
(per year longer)	0.98 (0.97–0.99)	<0.001	0.98 (0.97–1.00)	0.045
Drug or alcohol treatment ever				
(yes vs. no)	2.36 (1.51–3.68)	<0.001	2.33 (1.47–3.68)	<0.001
Any employment (reg, temp, self) ^a^				
(yes vs. no)	1.02 (0.74–1.42)	0.888		
Drug dealing^a^				
(yes vs. no)	1.90 (1.42–2.55)	<0.001	1.32 (0.96–1.83)	0.090
Sex work^a^				
(yes vs. no)	1.04 (0.67–1.61)	0.861		
High school degree or higher				
(yes vs. no)	1.15 (0.84–1.58)	0.379		
Overdose^a^				
(yes vs. no)	1.45 (0.85–2.49)	0.175		
Binge drug use^a,b^				
(yes vs. no)	1.76 (1.37–2.25)	<0.001	1.65 (1.26–2.16)	<0.001
A victim of violence^a^				
(yes vs. no)	2.19 (1.62–2.97)	<0.001	1.77 (1.29–2.42)	<0.001
HCV positive				
(yes vs. no)	0.97 (0.59–1.61)	0.916		
HIV and treatment status^a^				
(HIV+ and not on ART vs HIV−)	0.93 (0.59–1.46)	0.748		
(HIV+ and on ART vs HIV_HIV−)	0.76 (0.50–1.14)	0.186		
Incarceration^a^				
(yes vs. no)	1.68 (1.17–2.42)	0.005		

*GEE* generalized estimating equations, *PWID* people who inject drugs, *CI* confidence interval

^a^Denotes activities in the previous six months

^b^Refers to any route of consumption (i.e., sniffing, snorting, smoking or injecting)

**Table 3:** Treatment modalities participants were unable to access among PWID in Vancouver, Canada (*n* = 250)

Treatment modality	Number of reports	% of reports
Detox/youth detox	139	55.6
Treatment centre	38	15.2
Recovery House	33	13.2
Methadone or Suboxone	18	7.2
Counsellor	11	4.4
Daytox	6	2.4
Twelve-step programmes	3	1.2
Residential community	2	0.8
Out-patient treatment	2	0.8
Cocaine treatment program	0	0.0
Drug treatment court	0	0.0

*PWID* people who inject drugs

**Table 4:** Reasons for being unable to access addition treatment among PWID in Vancouver, Canada (*n* = 250)

Reasons	Number of reports	% of reports
Waiting list	146	58.4
Don’t take couples	20	8.0
Turned down by program	19	7.6
Personal reasons/issues	13	5.2
Communication issues with the program	11	4.4
Behaviour problems	9	3.6
Missed appointments	9	3.6
Program is full	8	3.2
Don’t know of any program	7	2.8
Don’t have type of program I want or need	7	2.8
Can’t afford the fees	7	2.8
Failed too many times	2	0.8
No treatment program nearby	1	0.4
Methadone restrictions within the program	1	0.4
Other (in jail, no pets policy, medical issues, too many rules)	39	15.6

*PWID* people who inject drugs

In the sub-analysis, the top three treatment modalities that participants were seeking but unable to access included inpatient detoxification services (66.4 %), inpatient treatment centres (14.8 %), and recovery houses (13.2 %), as shown in Table 3. Table 4 presents self-reported reasons for being unable to access addiction treatment. Being placed on a waitlist (58.4 %) was the primary reason participants gave for being unable to access addiction treatment, followed by the program not accepting couples (8.0 %) and being turned down by the treatment program (7.6 %).

## Discussion

We found that a substantial proportion of our study sample of PWID in Vancouver, Canada who were not enrolled in addiction treatment were unable to access addiction treatment despite motivated to do so. In the multivariate analysis, unsuccessful attempts to engage in addiction treatment were independently and positively associated with periods of homelessness, having ever been in drug or alcohol treatment, binge drug use, and reporting being a victim of violence, and were independently and negatively associated with length of time since initiating injecting drug use. The most common addiction treatment modalities reported to be inaccessible included inpatient detoxification, inpatient treatment centres, and placement at a recovery house. Though a variety of reasons were cited for inability to access addiction treatment, being placed on a waitlist, programs not accepting couples, and being turned down by program were the most common.

Our finding that PWID with longer injecting careers were less likely to experience inability to access addiction treatment has not been reported in previous studies, although a previous Australian study did find that long term drug users were more commonly in treatment [24]. These findings collectively demonstrate the need to ensure those individuals with shorter drug injecting histories have increased access to treatment in order to minimize future consequences of long term drug use, including declining health and increasing risk of death [25]. Additional studies understanding the differences in needs between individuals with variations in their length of injecting history may aid in addressing the barriers specific to newer users.

We identified a positive and independent association between an inability to access addiction treatment and binge drug use. This is particularly alarming as binge drug use has been identified as an independent risk factor of HIV seroconversion [22]. Somewhat surprising was our finding of an association between having ever been in drug or alcohol treatment and being unable to access addiction treatment, as this result has not been previously demonstrated. A negative association was previously identified between exposure to addiction treatment and attaining stable housing, suggesting that a history of addiction treatment may be a marker of severe drug addiction [26]. A recent Swiss study utilized an advanced statistical method to assess opioid agonist therapy utilization patterns and found that the time until readmission shortened as the number of treatment episodes increased [27]––a finding that somewhat contradicts our result. As cycling in and out of treatment is common among people with any substance use disorders, future research could apply such method to investigate patterns of participation in other treatment modalities, including detoxification services, and extend our finding. Regardless, it is essential that individuals who wish to enter treatment have the opportunity regardless of previous attempts.

We also identified a positive and independent association between being a victim of violence and reporting an inability to access addiction treatment. As previously reported, PWID are subjected to elevated levels of violence compared to the general population, commonly due to inextricable involvement in unpredictable drug market situations and informal activities, such as drug dealing, sex work and theft [28–30]. Individuals engaging in prohibited income generating activities also show more intense drug use patterns [31]. As a result of the violence experienced, many individuals will increase their drug use, experience physical injuries, and display an increase in mental health symptoms, all of which may have long term impacts on their health [32, 33]. Further, women who experience partner based violence often also have a lack of social support to actively pursue addiction treatment [34]. It is particularly concerning that those experiencing violence have difficulty accessing treatment, as this vicious cycle could be stopped by engaging PWID in addiction treatment to avoid partaking in risky drug use environments.

We also found that inpatient detoxification was the most common addiction treatment modality that participants were unable to access, with waitlists being the primary reason for this inaccessibility. This is consistent with previous studies showing that among referrals to a Vancouver-based in-patient detoxification, 35 % of clients dropped off the waitlist prior to commencing treatment [35]. Being placed on a waitlist has also been shown to decrease retention when in receipt of treatment, a problem which has been demonstrated not only in this setting, but other settings as well [35–37]. However, the criteria for requiring inpatient detoxification are evolving with stand-alone detoxification (without longer term outpatient treatment) being no longer advised in most cases of opioid addiction and in some cases of alcohol addiction [38, 39]. However, our findings do diverge from our past work that found disparities in access to addiction treatment based on ethnicity/ancestry [12], and is encouraging that we no longer find that people of non-Caucasian ancestry were more likely to experience difficulty obtaining addiction treatment, suggesting that recent efforts to scale-up access to treatment options, may be demonstrating positive results [10, 12]. In this particular setting in Vancouver, Canada, low threshold access to opioid agonist treatment is widely available through the universal no-cost medical insurance plan with the cooperation of community physicians and pharmacies. As a result of this integration participants would typically not have as much difficulty accessing opioid agonist treatment compared to other treatment modalities.

This study has several limitations. First, the VIDUS and ACCESS cohorts are not random samples and therefore may not generalize to other populations of PWID. Second, data collection was based on self-report and thus could be subject to reporting bias, including socially desirable responses which may have resulted in under-reporting of illicit drug use and other stigmatized behaviours. As a result, the prevalence of some risk behaviours may have been underestimated in the present study. However, self-reported risk behaviour has been shown to be largely accurate among adult drug-using populations [40]. Third, there were no variables representing family and social networking, which may have been important factors to analyze and should be included in future research. Lastly, as with any observational research, unmeasured confounders may exist that were not accounted for in our analyses and contributed to the overall results.

## Conclusion

In summary, despite the recent increasing support for addiction treatment in Vancouver [10], our findings indicate that some sub-populations of PWID are more likely to be marginalized from accessing addiction treatment services, including those who are homeless, those with shorter injecting careers, those who report binge drug use, those with previous alcohol or drug treatment experience, and those who report experiencing violence. Given that the primary reason we identified for inability to obtain addiction treatment was waitlists, it is imperative that additional resources go into ensuring treatment options are readily available when requested. Additionally, this study identified the need for targeted interventions for patient populations suffering severe negative consequences of their addiction as they are often the ones having a difficult time accessing treatment.

## Abbreviations

PWID: People who inject drugs
HIV: Human immunodeficiency virus
